# Stacked distribution models predict climate-driven loss of variation in leaf phenology at continental scales

**DOI:** 10.1038/s42003-022-04131-z

**Published:** 2022-11-10

**Authors:** Shannon L. J. Bayliss, Liam O. Mueller, Ian M. Ware, Jennifer A. Schweitzer, Joseph K. Bailey

**Affiliations:** 1 Department of Ecology & Evolutionary Biology, University of Tennessee, Knoxville, Knoxville, TN USA; 2 Department of Geography, Florida State University, Tallahassee, FL USA; 3 Department of Molecular Biology, University of California, San Diego, San Diego, CA USA; 4 Funga PBC 1612 Canterbury Street, Austin, TX 78702 USA

**Keywords:** Biodiversity, Ecological modelling

## Abstract

Climate change is having profound effects on species distributions and is likely altering the distribution of genetic variation across landscapes. Maintaining population genetic diversity is essential for the survival of species facing rapid environmental change, and variation loss will further ecological and evolutionary change. We used trait values of spring foliar leaf-out phenology of 400 genotypes from three geographically isolated populations of *Populus angustifolia* grown under common conditions, in concert with stacked species distribution modeling, to ask: (a) How will climate change alter phenological variation across the *P. angustifolia* species-range, and within populations; and (b) will the distribution of phenological variation among and within populations converge (become more similar) in future climatic conditions? Models predicted a net loss of phenological variation in future climate scenarios on 20-25% of the landscape across the species’ range, with the trailing edge population losing variation on as much as 47% of the landscape. Our models also predicted that population’s phenological trait distributions will become more similar over time. This stacked distribution model approach allows for the identification of areas expected to experience the greatest loss of genetically based functional trait variation and areas that may be priorities to conserve as future genetic climate refugia.

## Abstract (web summary)

Integrating stacked distribution models with genetically-based trait diversity of Populus angustifolia reveals how phenological variation can change differently across populations in response to climate change.

## Introduction

Human-induced environmental perturbations are expected to continue to limit plant population connectivity and gene flow, thus decreasing effective population sizes^1^ and ultimately reducing levels of genetic variation within plant species^2,3^. Maintaining genetic diversity within populations is essential for both short- and long-term survival of species facing rapid environmental change. Low levels of genetic variation reduce the ability of populations to adapt to novel conditions or to colonize new areas while simultaneously increasing the risk of bottleneck effects and inbreeding depression^4–6^. In addition to evolutionary consequences, loss of genetic variation will also have ecological consequences. Population genetic diversity, including variation in morphological, phenological, or resource-use traits can have strong effects on ecosystem functions^7,8^ such as biomass production and carbon cycling^9^ or nitrogen mineralization^10^. Further, experimental studies indicate that the positive effects of high genetic diversity may be most important for species’ responding to disturbed or stressful conditions^5,11–15^, like those expected with a changing climate.

Genetic diversity can refer to many forms of intraspecific variation. To predict the ecological consequences of diversity, it is critical to understand functional trait diversity, as functional traits of key organisms are determinants of ecological function^14,16^. For example, the genes of dominant species have been shown to have landscape-scale effects on ecosystem processes like carbon, nitrogen, and water cycling, and can alter resource availability for dependent organisms^7,10^. Though important, it is problematic that functional trait distribution has received little attention at broad spatial scales^17,18^. Though there has been an increase in research examining spatial genetic variation generally^19^, less is known about the ecological and evolutionary responses of functional traits to environmental change across species’ ranges or how traits may mediate or constrict population responses to climate change^16,20–22^. Some of the strongest responses to environmental change have occurred in traits like survival, fecundity, or phenology^23^ that influence many abiotic and biotic components of ecosystems^24,25^.

As species’ ranges contract, expand, or shift in response to climate change^17,26^ genetic variation is expected to be altered and/or reduced^19,20,27^. Plant population genetic diversity is related to biogeography such that core, and thus insular, populations often have the highest diversity^28^. Further, trailing edge (or “rear edge”) populations tend to have low population diversity while harboring unique diversity (i.e., regionally diverse) due typically to isolation and local adaptation^29^. Because plant population genetic diversity differs across space, so should our expectations of the ecological consequences of genetic diversity loss. Species distribution models are a useful tool for predicting species range dynamics in response to climate changes, but models are only beginning to include intraspecific variation^30^. While including population information is certainly a step forward in predicting range forecasts^31^, including functional trait information into these population models will allow for broader inferences about possible changes to ecosystem function.

Stacked species distribution models is an approach that has traditionally been applied to describe and predict species biodiversity patterns on the landscape^32^. The process “stacks” binary presence-absence maps built independently for multiple species to estimate biodiversity patterns across ecological gradients^32–34^. Here, we use this approach to instead model intraspecific trait variation across populations of a single species. This approach simultaneously tackled multiple issues in predicting species’ range shifts in response to climate change– Most models assume static trait-climate relationships which disregards differences in adaptive genetic variation on the landscape. This approach allows for populations with varying levels of genetic trait diversity to respond uniquely to climatic change. Equally important, many models of trait responses to climate change focus on shifts to population mean trait values rather than shifts to population trait variation. While shifts to population mean trait values are important indicators of evolution, they provide little information about populations’ ability to respond to selective pressures of changing climates. In this approach, a maximum trait richness prediction can reveal where on the landscape climate is not acting as a constraint to potential trait variation.

To address the challenge of predicting how genetically based trait variation may change on the landscape with climate change, we used three general circulation model predictions of future climatic conditions, genetically-based trait measurements of leaf out phenology of a dominant riparian species, *Populus angustifolia*, and the technique of stacked species distribution modeling^32^. Using these tools, we identify shifts in suitable climatic conditions for a range of phenological traits and ask the following questions: (1) how climate change will alter phenological variation across the species-range and within populations; and (2) if climate change will cause phenological trait variation to converge (become more similar) between populations. We hypothesized that changing environmental conditions associated with climate change will reduce phenological variation over the species-range, with the trailing edge (lowest latitude) population losing the most variation due to its already-limited distribution and position in the hottest and driest portion of the range. We also hypothesized that phenological trait distributions would become more similar within and among populations.

## Results

### Net loss of phenological variation in future climates

**Fig. 1:**
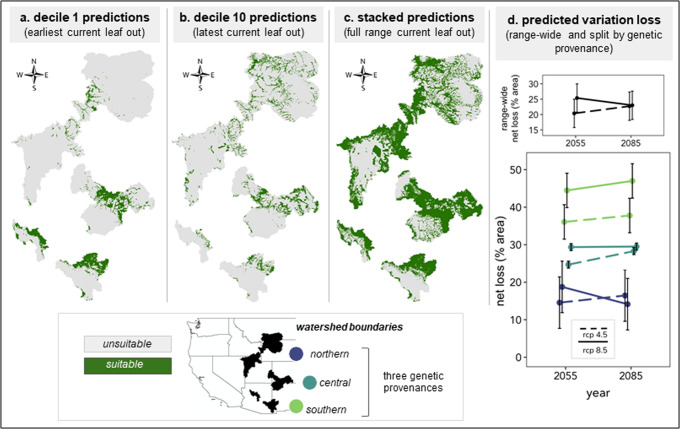
Maps of leaf out decile 1, leaf out decile 10, stacked deciles 1 through 10 as binary (presence-absence) predictions on current climate; Percent of the landscape predicted to lose phenological trait variation across three genetic provenances, two future time periods (2055, 2085), and two relative concentration pathways (RCP 4.5 and RCP 8.5). In **a**–**c**, shapes represent watershed designations for each genetic provenance, as described in the U.S. map; green represents suitable climatic conditions for the modeled trait decile (or deciles) and gray represents unsuitable climatic conditions for the modeled trait decile (or deciles). In **d**, “net” percent loss is calculated as the difference between percent loss and percent gain. Points (+/−standard error bars) represent average predictions from three Atmosphere and Ocean General Circulation/Climate models (AOGCMs) that capture “best”, “worst”, and “median” climate projections for this geographic region. Color represents genetic provenance of *P. angustifolia*, as labeled in **c**. Yellow green represents the southern provenance, green blue the central provenance, and blue purple the northern provenance. Line type (dashed or solid) represent RCP 4.5 and 8.5 respectively. For visual clarity, this map does not show occurrence data. These data, with latitudes and longitudes, can be found in the supplementary information.

**Fig. 2:**
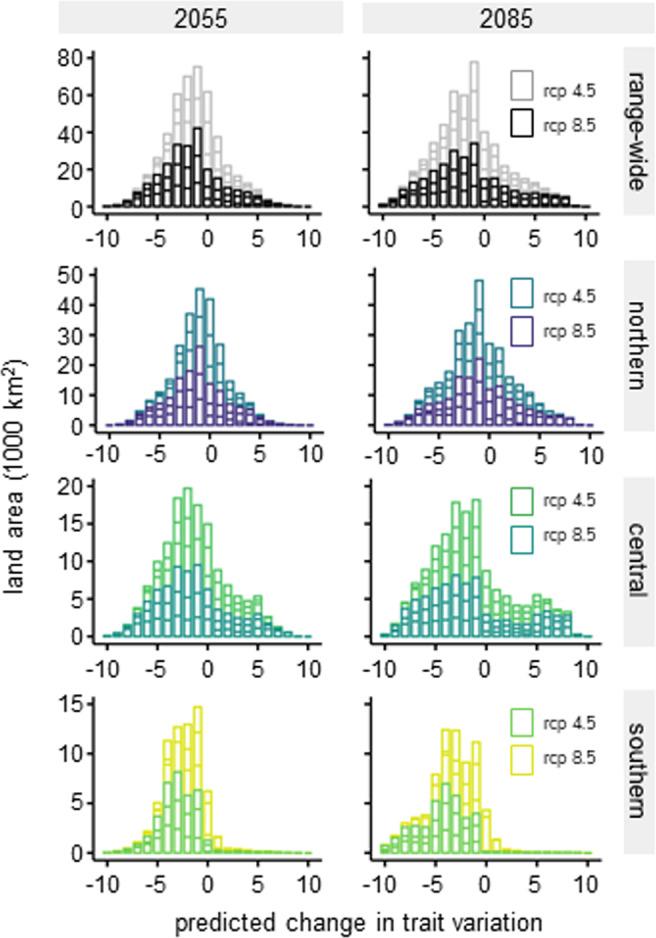
Predicted changes in phenological trait variation (decile richness) on the landscape (area) across the *P. angustifolia* species range, three genetic provenances, two future time periods (2055 and 2085), and two relative concentration pathways (rcp 4.5 and 8.5). The lighter color lines within each bar represents rcp 4.5 predictions while the darker color within each bar represents rcp 8.5 predictions. Lines across each bar represent the predictions made from each of the three Atmosphere and Ocean General Circulation/Climate models (AOGCMs) for each time and rcp. Zeros only represent no change in trait variation if there was variation to begin with (i.e., they do not represent area of unsuitable climatic conditions). Positive values indicate gains in trait variation.

With all examined future climate scenarios, approximately 20-25 percent of the total landscape modeled was predicted to have a net loss of trait richness (Fig. 1 and Table 1), indicating reductions in genetic variation for foliar leaf out. This net percent loss decreases with latitude and across genetic population, such that the southernmost population is predicted to lose phenological traits richness on the largest amount of land – between 36.1% (rcp 4.5, 2050 s) – 47.0% (rcp 8.5, 2080 s), while the northernmost population is predicted to lose phenological trait richness on the least amount of land area, between 14.1% (rcp 8.5, 2080 s) and 18.8% (rcp 8.5, 2050 s). The central population is predicted to lose phenological trait richness on 24.7% (rcp 4.5, 2050 s) to 29.5% (rcp 8.5, 2080 s) of the landscape (Fig. 1, Table 1, and Supplementary Fig. S1). We found significant differences between genetic populations but that these differences did not vary with relative concentration pathways or year (Fig. 1 and Supplementary Fig. S1; *F*_population_ = 27.9, *p*_population_ < 0.001). However, the *total* percentage of landscape predicted to lose phenological trait richness (i.e., without adjusting for trait richness gains) did change significantly across genetic population and with relative concentration pathways (*F*_population_ = 36.8, *p*_population_ < 0.001; *F*_rcp_ = 7.8, *p*_rcp_ = 0.01; *R*^2^ = 0.78). Losing suitable climatic conditions for one decile in a location is very different from, for example, losing suitable climatic conditions for eight deciles in the same location. For this, it is important to not just quantify positive and negative losses across space, but also the degree of change in trait variation. Following the predictions made of landscape-level net losses across populations, sites in the southernmost population are predicted not only to lose the most suitable climatic conditions overall, but also to lose the highest number of trait deciles regardless of time or relative concentration pathway (Fig. 2). In contrast, shifts in the number of trait deciles lost for the central and northernmost populations are countered more by gains, suggesting phenological distributional shifts occurring in some areas, despite net loss of variation in foliar phenology (Fig. 2).

**Table 1:** Net loss of foliar phenology trait variation calculated as percent loss minus percent gain and 95 percent confidence bounds averaged for the three global circulation models across geographic range (genetic provenance), year, and relative concentration pathway (RCP).

Geographic extent	Year	Net loss (%) [lower CI, upper CI]	
		rcp 4.5	rcp 8.5
Species range	2050s	20.4 [9.8, 31.0]	25.4 [14.8, 36.0]
	2080s	22.7 [12.1, 33.3]	23.0 [12.4, 33.6]
Northern	2050s	14.6 [−1.3, 30.4]	18.8 [2.9, 34.6]
	2080s	16.5 [0.6, 32.3]	14.1 [-1.7, 30.0]
Central	2050s	24.7 [22.6, 26.8]	29.4 [27.2, 31.5]
	2080s	28.3 [26.1, 30.4]	29.5 [27.4, 31.6]
Southern	2050s	36.1 [25.5, 46.6]	44.5 [33.9, 55.0]
	2080s	37.8 [27.2, 48.4]	47.0 [36.4, 57.6]

### Phenology richness patterns from stacked distribution models

Each phenology decile model predicted suitable (current) climatic conditions on 10.7 to 33.2 percent of the landscape (Table 2). The first and tenth deciles, which represent the earliest and latest leaf out times, as well as the largest range of leaf out days overall (as the two tails of the trait distribution) predicted the least suitable area overall (10.7 and 11.5%, respectively; Table 2). The third decile predicted the most suitable area at 33.2 percent. Interestingly, this is almost the full area predicted suitable by stacked decile models: 34.3 percent (Table 2). Only 2.8 percent of the land area was predicted to have suitable climatic conditions to support the full range of phenological trait values (i.e., all ten trait deciles: Fig. 1a).

**Table 2:** Summary and evaluation of decile distribution models.

Modeled trait decile	^N^occ	Predicted suitable		Model evaluation		
		Pixel	% area	^AUC^test ± ^sd^	^AUC^train	Omission rate
D1	39	16,840	10.7	0.797 ± 0.090	0.950	0.077
D2	46	47,386	30.2	0.875 ± 0.056	0.933	0.043
D3	41	52,064	33.2	0.845 ± 0.071	0.913	0.098
D4	39	41,900	27.2	0.884 ± 0.049	0.920	0.077
D5	38	49,687	31.7	0.812 ± 0.067	0.896	0.079
D6	39	32,359	20.6	0.848 ± 0.050	0.911	0.077
D7	39	40,574	25.9	0.831 ± 0.050	0.900	0.077
D8	44	29,752	19.0	0.886 ± 0.042	0.940	0.091
D9	41	32,326	20.6	0.856 ± 0.052	0.902	0.098
D10	34	17,959	11.5	0.879 ± 0.049	0.943	0.088
Stacked Models: 400		53,727	34.3			

Predicted suitable climatic conditions on the landscape are listed both as pixel number and as percent area of the total extent of the landscape (total pixels = 156,659). Each pixel has an area of ~1 km^2^, a resolution of 30 arc seconds. Test AUC (area under the receiving operating characteristic curve) +/−standard deviations are given from 5-fold cross-validation. Training AUC is for final models. Training omission rate is for the 10-percentile training presence threshold rule.

### Phenological trait distributions shift and converge

**Fig. 3:**
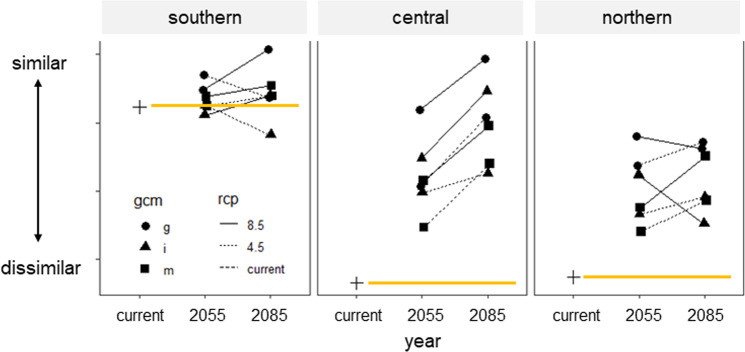
Changes in similarity for phenological trait distributions across time and relative concentration pathways for each of three genetic provenances of *P. angustifolia* as predicted by three Atmosphere and Ocean General Circulation/Climate models (AOGCMs). Each point represents an average similarity value for each general circulation model abbreviated as “i” (inm-cm4), “m” (mpi-esm-lr), and “g” (gfdl-cm3). Higher similarity represents a narrow range of phenological trait values. The yellow lines extend from the baseline similarity within each provenance as predicted on climate norms (i.e., extended from the “current” value indicated by the “+” sign). Values above the yellow line indicate that trait values are becoming more similar over time.

We examined phenological distribution change within populations by comparing predictions made by future climate scenarios to the current (“baseline”) distribution of traits. Within each population, the distribution of phenological variation becomes more similar from the original state (Fig. 3). With few exceptions, most AOGCM and RCPs predicted more similar phenological trait distributions through time (i.e., points above yellow line in Fig. 3). Overall, the southern population is predicted to see the smallest changes to phenological trait distributions, and the central and northern populations are predicted to have greater changes in phenological distribution (Fig. 3). Genetic population explained 51.9% of the variation in trait distributions, or community composition of trait deciles, to continue the community ecology analogy, across the species range (RDA; compared to a null model *p* < 0.001). The best model included the global change model (AOGCM) in addition to genetic population as constrained variables, explaining 64.7% of the variation. This addition is expected given that the global change models were chosen to represent different future climatic conditions in the study area. Though distributions of phenological trait variation are predicted to change within all three genetic populations, the effect of relative concentration pathways on the degree of change appears variable, suggesting that significant losses and shifts will occur even in lower emissions scenarios.

**Fig. 4:**
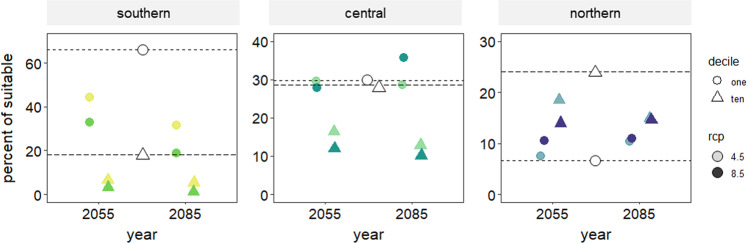
Average projected change in frequency of the earliest 10% (decile one) and latest 10% (decile 10) of leaf out phenology across three genetic provenances of P. angustifolia relative to the total landscape with at least a richness value of 1 (i.e., excluding unsuitable area) across genetic provenances. Empty symbols and dotted lines represent climate norms or starting conditions and filled symbols represent frequencies at each time averaged across AOGCMs. Circles represent decile 1 and triangles represent decile 10. Lighter shades represent RCP 4.5 and darker shades represent RCP 8.5.

We also examined the average frequencies of the earliest (decile 1) and latest (decile 10) 10% of individuals to leaf out across time relative to the amount of landscape projected to have suitable climates for at least one trait decile (i.e., landscape that was predicted to have a richness of 1 or greater). The southern population was modeled to currently contain a higher frequency of early foliar phenology relative to later phenology, and the northern population with a higher frequency of later, relative to early phenology (Fig. 4; also Fig. 1a, b). The central population began with a similar frequency of both deciles (Fig. 4). Across time, the loss of early and late phenology in the southern population is proportional to the beginning frequencies (Fig. 4). The central population loses late phenology trees while the northern population loses late phenology and gains early phenology trees (Fig. 4).

### Model reliability and environmental contributions to models

Distribution models for each trait decile had good predictive accuracy with an average test AUC of 0.85 (Table 2 and Supplementary Fig. S2). Stream order contributed consistently high (between 30-54 percent) to all modeled trait deciles (Supplementary Fig. S2). Climatic moisture index contributed significantly more to later decile models (Supplementary Fig. S2; *R*^2^ = 0.68, *p* = 0.0031) while winter precipitation and relative humidity (Supplementary Fig. S2) contributed significantly more to earlier phenological trait decile models (respectively, *R*^2^ = 0.53, *p* = 0.017; *R*^2^ = 0.49, *p* = 0.024). Continentality contributed between 1.5 to 22 percent to the ten models but showed no significant relationship in its importance across decile models (Supplementary Fig. S2). These significant relationships reflect environmental variable contribution to model predictions, and thus should not be interpreted as a description of the variable’s relationship to trait values. For example, earlier leaf out was related to higher climatic moisture index, higher winter precipitation, lower relative humidity, and lower continentality, though these specific relationships varied by population (Supplementary Fig. S3).

## Discussion

To our knowledge we are the first to take a stacked distribution modeling approach to map the potential distribution of a genetically diverse functional trait (i.e., foliar phenology) on the landscape. Despite current theory moving towards understanding how climate exerts selection on the landscape, we have only recently begun collecting sufficient data to support these hypotheses (e.g., Ware et al. 2019^35^). This lack of data and approaches by which the relationship between climate change and genetic variation in functional traits can be examined is significant. By integrating phenological trait variation of 400 *Populus angustifolia* genotypes with stacked species distribution models, our results predict a large loss of phenological trait variation across the *P. angustifolia* species’ range in response to future climatic conditions (i.e., selection for specific phenology dates). Moreover, models predict overall that convergence among populations in phenological trait distributions will occur irrespective of emission scenario (i.e., reducing genetic variation for phenology will lead to more similar ranges of phenology traits). These results have important implications for the ability of populations to respond and persist in the face of ongoing climate change, the potential of those populations to support associated species that depend upon them as food and habitat, and for the potential of climate- driven ecosystem disassembly due to predicted changes in total productivity resulting from changes in total growing season length.

Species can go extinct, shift their distribution, adapt, or acclimate in response to a changing climate. All potential responses depend upon trait variation^36^, yet most research on trait responses to climate change focus on shifts to mean trait values of populations rather than shifts to trait variation of populations^37^. While shifts among population mean trait values are important indicators of evolution on the landscape, they provide little information or context into how those populations may respond to selective pressures exerted by changing climates in the future. Though we don’t explicitly examine individual climate gradients that could be driving these patterns, previous work in this system demonstrates that broad-sense heritability in leaf out phenology decreases (less genetic trait variation) across a gradient of increasing temperature^35^. Overall, our models predict a net loss of genetic variation for foliar phenology across all *P. angustifolia* riparian forest populations in the western US if these mature adult trees do not survive. The loss of genetic variation occurs irrespective of emission scenario and could be seen as soon as the 2050 s. Losses of genetic variation will be greatest in the trailing edge (low latitude) population – occurring on up to 47% of the landscape (Fig. 1d). In general, however, trailing edge populations tend to have low trait diversity, but they also tend to be regionally diverse due to isolation and/or strong local adaptation^29^.

Models also predicted that traits distributions will homogenize between the three genetic populations over time (Fig. 3b; Fig. 4), a result that has been empirically demonstrated across elevation for four European temperate trees^24^. The convergence of all populations on a similar functional trait distribution is concerning as it suggests that the ability of individual populations to respond uniquely to climatic changes may be constrained in future climate scenarios. Variation is critical for evolutionary response to environmental change. Importantly, the predictions made from these models occur regardless of RCP (4.5 and 8.5), period (2050 s and 2080 s), and AOGCM, suggesting that regardless of climate scenario, the changes are predicted to occur by mid-century.

It is increasingly clear that genetic variation in functional traits is critical in mediating patterns in biodiversity and ecosystem functions^7,8,38^. This is especially true for dominant species like *P. angustifolia* whose genes have been shown to have wide-ranging, landscape-scale effects on the land-atmosphere and plant-soil linkages and feedbacks affecting the carbon and nitrogen cycles^7,35,39^. For example, changes to plant phenology can affect growing season length, and thus biomass production and carbon cycling^35,40,41^, evapotranspiration, stream discharge^42,43^, and resource availability for dependent organisms^25^. Phenology can also influence soil microbial community structure and function in a way that can feedback to affect plant performance under climate change^44^. If one consequence of climate change is the general reduction of genetic variation in functional plant traits, then there will be cascading effects on other biodiversity and ecosystem function.

The relationships between biodiversity – including genetic – and climate are critical for conserving natural ecosystems and their functions^45^. Climate relict populations have persisted under a changing climate and are largely considered “life-boats” for associated species and ecosystem functions^46^. Cottonwood riparian forests of the western US are already rare, estimated to occupy less than 5% of the landscape, and yet support most of the biodiversity of the west^47^. Our previous research indicates that trailing edge population of *P. angustifolia* are locally adapted in functional traits related to water and carbon cycling that drive land-atmosphere feedbacks^48^. Further, genotypes from the trailing-edge population are an important source of heat and water stress-tolerant individuals, making them critically important for conservation. If the loss of genetic variation in these populations is non-random (i.e., driven by selection) as our models suggest, and genetic variation in functional traits supports variation in ecosystem functions on the landscape^22,35,48^, then climate driven loss of genetic variation may be an important driver of ecosystem disassembly.

In this work, we adapt stacked species distribution modeling, a method of species distribution modeling that has only been applied to models of species’ diversity, to model genetically based diversity of spring leaf phenology. This approach simultaneously tackles multiple issues in predicting species’ range shifts in response to climate change. Most models assume static trait-climate relationships, or niche conservatism, consequently disregarding the potential for populations with varying levels of genetic trait diversity to respond uniquely to climatic change. Additionally, and related, models of trait responses to climate change focus on shifts in population mean trait values rather than shifts in population trait variation. While shifts to population mean trait values are important indicators of evolution, they provide little information about a populations’ ability to respond to selective pressures of changing climates. Foliar phenology has important implications for total ecosystem productivity. Results from this novel application of stacked SDMs predict shorter and earlier leaf out seasons that will likely have implications for whole ecosystem dynamics.

## Methods

### Study species, occurrence data, and geographic extent

*Populus angustifolia* James is a dominant riparian tree species distributed from the south of Alberta, Canada along the U.S. Rocky Mountains and into the north of Chihuahua, Mexico^49^ spanning approximately 1700 km of latitude. The species exhibits a wide range of physiological, growth, and phenological trait variation across this large geographic range and gradient of climates^35^ and is thus ideal for examining how genetic variation for functional traits are distributed on the landscape and may shift in response to climate change.

Tree occurrence data were collected in the field for *Populus angustifolia* in May-June 2012 (Supplementary Fig. S4). Latitude and longitude coordinates were collected as decimal values from the WGS 84 world grid system for each sampled tree. Occurrence data span the range of three known genetic populations (Arizona, Eastern, Northern/Wasatch Clusters)^50,51^, which we will refer to as the “southern”, “central” and “northern” populations, for ease of communication and map visualization (Supplementary Fig. S4). Sampling covered extreme environments as well as locations near the edges of the species’ geographical range, which should provide comprehensive predictions of range dynamics expected with climate change^29,52–54^. The original occurrence dataset included 725 individual geo-referenced trees, but not all trees survived for greenhouse phenology data collection (see below, 400 genotypes survived to have phenology measurements made, and these field locations are represented in Supplementary Fig. S4). Cuttings were made from each field tree and replicates of each genotype were grown under common conditions at the University of Tennessee. See Ware et al. 2019^35^ for details regarding the establishment of greenhouse plants.

When building distribution models, the geographic extent selected to train the model should be based on assumptions about species’ dispersal. With *Populus*, genetic connectivity of populations has been shown to be related to riparian network connectivity^54,55^, and thus we chose to create geographic bounds for training our models with water basin designations. Based on the field locations of collected trees, we created boundaries for model training with the smallest watershed subdivision from the USGS Watershed Boundary Dataset that captured all occurrence coordinates. This led us to choose HUC (hydrologic unit code) level 6 (https://water.usgs.gov/GIS/huc.html).

### Phenology data

Tree cuttings were taken from the same individuals during 2012 when occurrence data were collected. These tree cuttings were grown for four years in a greenhouse at the University of Tennessee. In 2016, phenology was measured for these tree cuttings as the day the first leaf unfurled on each plant in the spring. This determined the genetic basis of phenology since all trees were growing in the same common environmental conditions. Measurements were made on 400 genotypes from across the species’ range (the original dataset had 725 genotypes). Of this 400, 57 genotypes were from the southern population, 157 were from the central population, and 186 were from the northern population. The earliest observed leaf out was recorded on Julian day 71 (March 11^th^) and the latest observed leaf out was recorded on Julian Day 125 (May 4^th^), which resulted in a 54-day range across which genetically based leaf out occurred (Supplementary Fig. S3); 2016 was a leap year. Because our SDM methodology relies on binary presence-absence data, we subset the dataset (paired phenology measurements (which determine the deciles) with georeferenced field location of the parent tree) into ten smaller datasets based on deciles of the trait distribution, each part representing one-tenth of the total sample. For example, the first subset/decile represented the first 10% of trees that leafed out. Ten was chosen so that the sample size of each model would be around 40, which is above the minimum records (20-30) suggested to accurately evaluate model performance^56^. The exact number of points for each decile model varied slightly due to spatial thinning (i.e., so no single grid cell would hold more than one presence point for a given decile model, eliminating any pseudo-replication) and so that no trait values fell within more than one decile. The associated field coordinates for each decile were used to build separate species distribution models to make predictions about the effects of climate change on phenological trait variation (see below section “*Species distribution model calibration and evaluation”)*. The southern population was the only population that did not have trait values in all ten deciles (missing the 7^th^, 8^th^, or 10^th^ deciles).

### Environmental variable selection

We obtained bioclimatic variables from the AdaptWest Project^57^, and stream order (Strahler) was obtained from the National Hydrography Dataset to constrain the riparian habitat of *P. angustifolia* (NHDPlusV2)^58^. NHDPlusV2 is a companion dataset to the USGS Watershed Boundary Dataset from which we derived our watershed regions. All variables were the same spatial resolution (30 arc second; ~1 km at the Equator) and were projected into the same coordinate system (WGS1984) in ArcMap^59^. Based on a previous set of species distribution models built to describe the species’ range^60^, we selected a subset of the 27 available climatic variables. This is based on common practice of using expert judgment on the ecology of the taxa^61^ to reduce multi-collinearity and over-fitting of models^62^. From our previous models describing population and species distributions, we selected those variables that had contributed at least 10% to model predictions, resulting in a subset of ten variables. These variables had already been tested for spatial correlation. AHM: annual heat moisture index; CMD: Hargreave’s climatic moisture index; DD_0: chilling degree days/degree-days below zero °C; Eref: Hargreave’s reference evaporation; MAP: mean annual precipitation; NFFD: number of frost-free days; PAS: precipitation as snow; PPT_wt: winter (Dec-Feb) precipitation; RH: relative humidity; and TD: difference between mean temperature of the coldest and warmest months, as a measure of continentality.

Final selection of variables was made from an initial SDM run for all ten phenology (i.e., day of year) decile models using the initial subset of variables described above. We summed the contributions of individual variables across the ten decile models and eliminated those that did not contribute highly to any decile models. Note that because we expect individual decile models to have different relationships to these predictor variables, we do not expect that the final contributions of the selected variables will be equally high across the ten decile SDMs. Variables maintained for final model runs included: Hargreave’s climatic moisture index, winter (Dec-Feb) precipitation, relative humidity, and continentality. Finally, stream order was included with all models and model projections to constrict the predictions to riparian zones. Variation in these variables is visualized in Supplementary Fig. S5.

To reveal the relationship between environmental variables and phenology, we ran linear mixed effects models in R with the package “lme4”^63^ using continuous leaf out data (days, not split into deciles) as the response variable, after checking for normality. We extracted values of the five environmental variables used in the distribution models for the field locations of our genotypes and used these variables as predictor variables with population as a random effect. All models had lower AIC values when population was included as a random effect, compared to null models (Supplementary Table S1). Results and figures from these models can be found in the supplementary materials.

### Species distribution model calibration and evaluation

We used MaxEnt software to build ten species distribution models (Version 3.3.3k)^64^. MaxEnt is widely accepted to perform well with low sample size and with presence-only data^65,66^. Each model was trained with one-tenth of the species’ occurrence data corresponding to phenological trait deciles, measured in the greenhouse. For example, the associated coordinates of origin for the first ten percent of greenhouse trees that broke bud in the spring of 2016 were modeled as “one species” for the purposes of “species” distribution modeling. This example model is referred to as the “decile 1 model” in the manuscript (and accordingly for the 2^nd^, 3^rd^, etc. deciles). Each MaxEnt model was formatted to run with logistic output for best conceptualizing the output as estimates of the probability of suitability between values of 0 (unlikely to be present) and 1 (likely to be present). For each decile model, we ran 5-fold cross-validation and evaluated their performance with area under the receiver operating characteristic curve (AUC) metric. An AUC value of 1 indicates perfect discriminatory ability, while a value of 0.5 indicates random predictions. Each of the distribution models had good predictive accuracy with an average test AUC value of 0.85, and values ranging from 0.79 to 0.89 (Table 2)^63,67^. Analysis was repeated using all occurrence data to train final models for each trait decile, against ~10,000 background points with 340-500 iterations.

To obtain binary output to test our hypotheses, we applied a 10% training presence threshold rule. This threshold rule finds the suitability value at which 10% of the training presence points are predicted absent (i.e., omission error) and uses it to reclassify pixels with suitability values below that value as unsuitable (absent) and above as suitable (present). It should be noted here that each decile model ended up having slightly different sample sizes which does introduce variability in sizes of training and testing subsets (Table 2): this is partially due to the model omitting occurrence points if they fall within the same pixel as another point, and partially due to each unique trait value only being assigned to a single decile.

### Using stacked distribution models to estimate effects of climate change on phenological trait variation

To estimate changes in genetically based variation in plant phenology on the landscape, we applied the principle of stacked species’ distribution modeling to binary maps of the ten trait decile models. Stacked SDMs sum (“stack”) binary presence-absence maps built independently for multiple species to estimate species richness on the landscape^32^. Typically, this method has been used to estimate biodiversity patterns across ecological gradients (e.g., elevation^32,33^) or to make predictions of biodiversity change with climate change^68,69^. Stacking ten decile models allowed us to derive what is analogous to species richness – essentially “decile richness” – or the total number of trait deciles predicted at a given location. This metric describes trait variation in a location on the landscape. A maximum “decile richness” of ten indicates that phenological trait variation is genetically unconstrained at that location: or, in other words, that all observed values of the trait could exist in the climatic conditions at that geographic location.

To test hypotheses about how future climate will affect phenological trait distributions, we projected each of the ten decile models into 12 future climate scenarios [3 general circulation models x 2 relative concentration pathways x 2 time periods], resulting in 130 distribution maps. Future climate data was from ClimateNA AdaptWest Project, with the Coupled Model Intercomparison Project Phase 5 (CMIP5) database derived from the 5^th^ IPCC assessment report (AdaptWest Project 2015)^57^. We selected three Atmosphere and Ocean General Circulation/Climate Models (AOGCMs) to capture the “best case,” “median case,” and “worst case” projections in the geographic range of interest including the U.S. states of New Mexico, Arizona, Colorado, Wyoming, and Utah where the rivers are located. We chose this approach to represent the extremes of potential future scenarios in this region, rather than selecting ensemble models that average algorithm predictions of future climates. For geographic region, we selected INM-CM4, MPI-ESM-LR, and GFDL-CM3, all of which have high validation statistics^70,71^, from which we downloaded data for representative concentration pathways (RCP) 4.5 and 8.5 (to represent a better and worse case scenario) for time periods 2055 and 2085.

For each of the 12 future climate scenarios we again stacked models to calculate predicted “trait richness” as a measure of trait variation (i.e., phenological variation) on the landscape. To calculate the change in trait richness on the landscape we subtracted current trait richness values from projected richness values for each grid cell: resulting in values from -10 to 10. Because zeros could indicate no change in trait richness (with or without decile composition changes) or unsuitable climatic conditions for all trait deciles, we re-calculated trait richness change considering which pixels were unsuitable when modeled with current conditions. We calculated net change as the difference between the percent of the landscape predicted to experience trait richness losses and gains, regardless of the degree of change (i.e., losses of 2 or 7 are both considered a loss). Stacked SDMs tend to over-estimate species richness^58^ and thus, in our study, results of “richness loss” may be conservative. To examine whether predicted changes in trait richness due to climate change would differ across genetic populations of *P. angustifolia*, we repeated calculations of trait richness change within each watershed that contained occurrence points from the three genetic populations. We tested whether the net amount of landscape predicted to lose trait richness differed across populations, years (current, 2055, and 2085), and emissions scenarios with linear regression models (lm function of R^72^).

### Estimating phenological trait distribution similarity over time within and among genetic populations

To estimate how frequent different combinations of trait values are, and how these frequencies are liable to change with future climate relative to their initial combinations, we constructed matrices for each genetic population with rows representing climate scenario and columns representing all possible combinations of deciles predicted to exist together on the landscape (i.e., 1023 possible combinations of ten numbers are possible). This is closely analogous to an abundance “site” (climate scenario) by “species” (decile combinations) matrix, as used in community ecology. Instead of a community of species, we have communities of trait deciles. Values in the matrix represent pixel counts of suitable climatic conditions for that combination of trait deciles. There are 45 different combinations of a “decile richness” of 2, however, a 2-decile combination of decile 2 and decile 5, for example, is a “rare community” relative to a 2-decile combination of deciles 2 and 3, or deciles 4 and 5. To account for this, we calculated a similarity score as an average distance between all observed trait deciles in a cell. To compare changes within populations, we compared future “sites” to the current baseline “site” composition. Increases would indicate convergence of trait communities and decreases would indicate divergence of trait communities. We used redundancy analysis to quantify the amount of variation in trait distributions explained by genetic population using the function “rda” from the “vegan” package^73^. Additionally, we tested the inclusion of all factors as constraining variables in the RDA using the function “ordistep” which performs backwards and forwards model selection using permutation tests.

## Supplementary information

Supplementary Information-New

Description of additional supplementary files

Supplementary Data 1

Supplementary Data 2

Supplementary Data 3

Supplementary Data 4

Supplementary Data 5

Supplementary Data 6

nr-reporting-summary

## Data Availability

Data for all manuscript figures are available as Supplementary Data 1–6. Other information generated during the current study that are not included in the manuscript are available from the corresponding author upon reasonable request.

## Code availability

Codes were written and performed in R and are available from the corresponding author upon reasonable request.
