# Supplementary material for: Stacked distribution models predict climate-driven loss of variation in leaf phenology at continental scales
Source: Commun Biol. 2022 Nov 10;5:1213. doi: 10.1038/s42003-022-04131-z (PMC9649771; doi:10.1038/s42003-022-04131-z)
Supplement: Supplementary file 1 — Supplementary Information-New [file 42003_2022_4131_MOESM1_ESM.pdf]

Supplemental Information for: Stacked distribution models predict climate-driven loss of variation in leaf phenology at continental scales

Authors: Shannon L.J. Bayliss*[1,2], Liam O. Mueller[3], Ian M. Ware[4], Jennifer A. Schweitzer[1], Joseph K. Bailey[1]

*Corresponding author; shannonljbay@gmail.com

Includes:

Supplementary Table 1

Supplementary Figures 1-5

Supplementary Table 1 (Table S1): Comparison of model AIC values for linear mixed effects models with and without population as a random effect. Individual models had each environmental predictor variable from SDMs and genetic population as predictors of leaf-out day. Models from this table correspond to panels f-j of supplemental figure 3.

Environmental Predictor	AIC Full Model (Population as random effect)	AIC Null Model (No random effect)
Stream order	2875.85	3000.70
Climatic moisture index	2884.27	2966.17
Winter precipitation	2869.21	2992.01
Relative humidity	2847.05	2972.10
Continentality	2839.56	2975.41

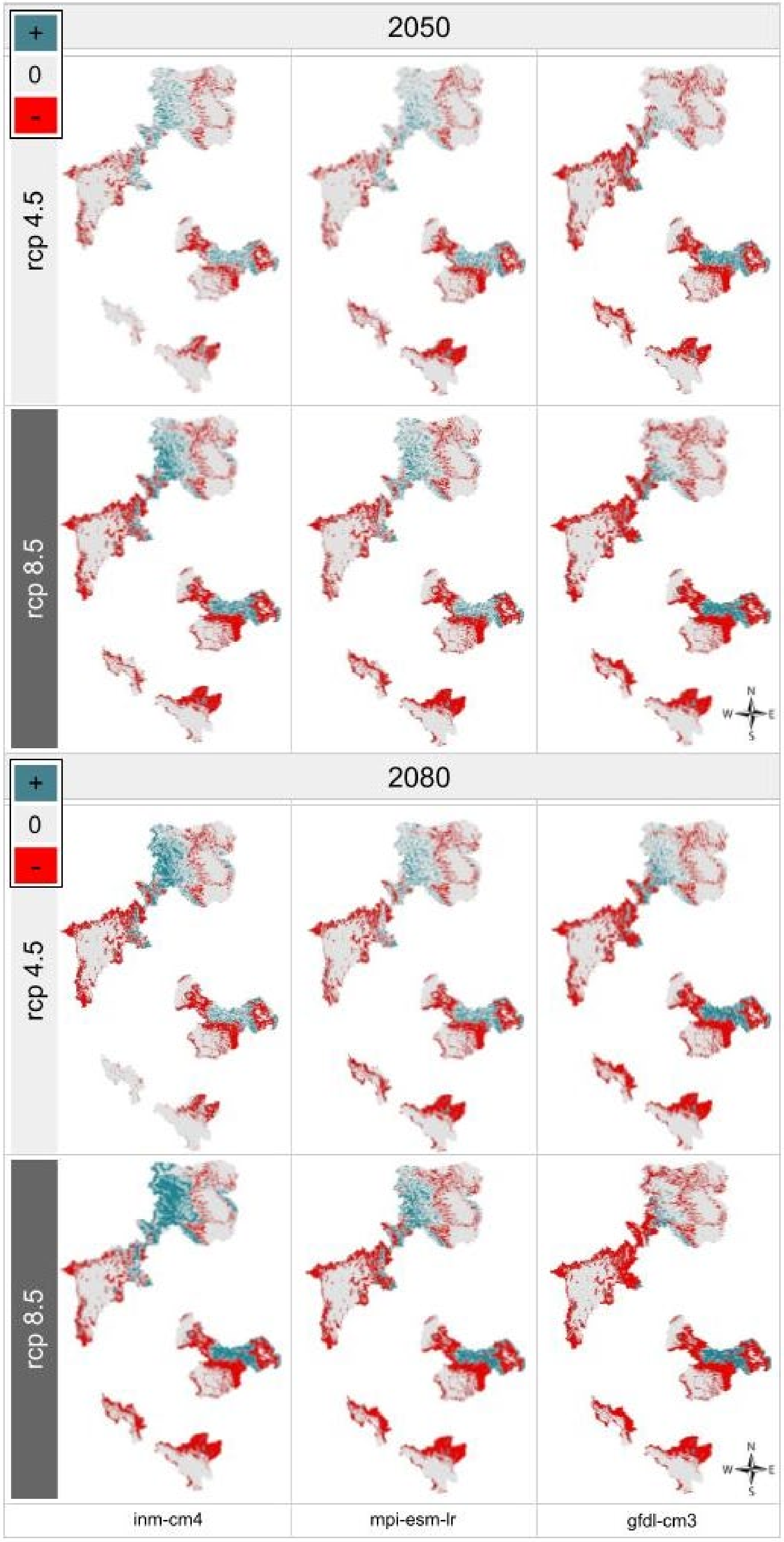
2050
+
0
-
rcp 4.5
rcp 8.5
N
W
E
S
2080
+
0
-
rcp 4.5
rcp 8.5
N
W
E
S
inm-cm4
mpi-esm-lr
gfdl-cm3

Supplementary Figure 1 (Fig. S1): Maps of richness loss and gain by two future time periods (2050s and 2080s), two emissions scenarios (rcp 4.5 and rcp 8.5), and three Atmosphere and Ocean General Circulation/Climate models (AOGCMs; inm-cm4, mpi-esm-lr, and gfdl-cm3). Areas predicted to gain trait richness are represented in blue. Areas predicted to lose trait richness are represented in red. Grey areas are areas where no change in richness is predicted.

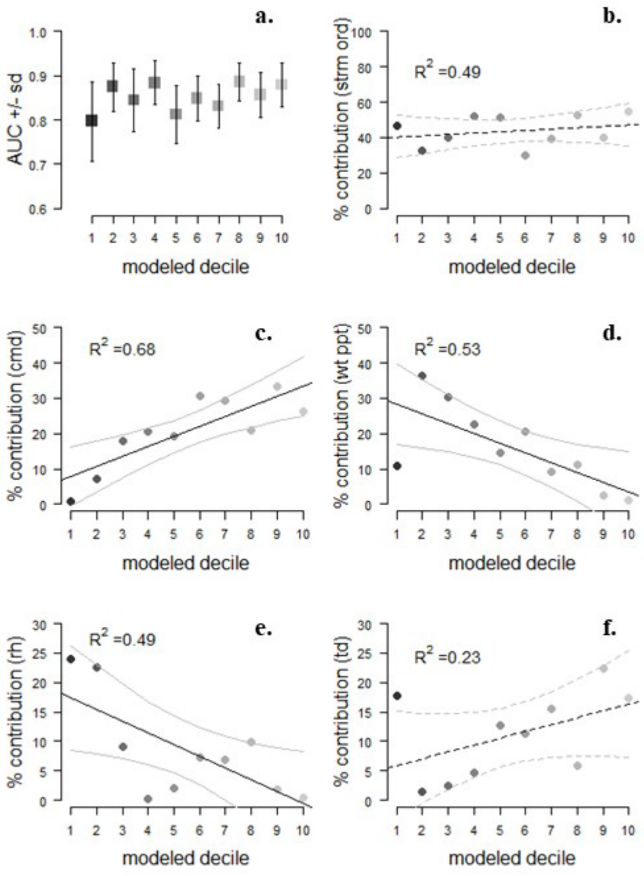

Supplementary Figure 2 (Fig. S2): Model performance and environmental variable contributions to leaf out decile distribution models. (a) represents the average test area under the receiver operating characteristic curve (AUC) +/- standard deviation from 5-fold cross-validation of decile models. An AUC value of 1 indicates perfect discriminatory ability, while a value of 0.5 indicates random predictions. Panels **(b-f)** represent the percent contributions of each environmental variable to the predictions made by decile models: **(b)** Strahler stream order, **(c)** Hargreave's climatic moisture index in mm, **(d)** winter precipitation in mm, **(e)** percent relative humidity, and **(f)** continentality, measured as the difference between mean temperature of the coldest and warmest months in degrees Celsius. Note the different scales of y-axes. Solid lines of confidence intervals represent significant linear relationships at a threshold of p=0.05 and dotted lines of confidence intervals represent non-significant relationships.

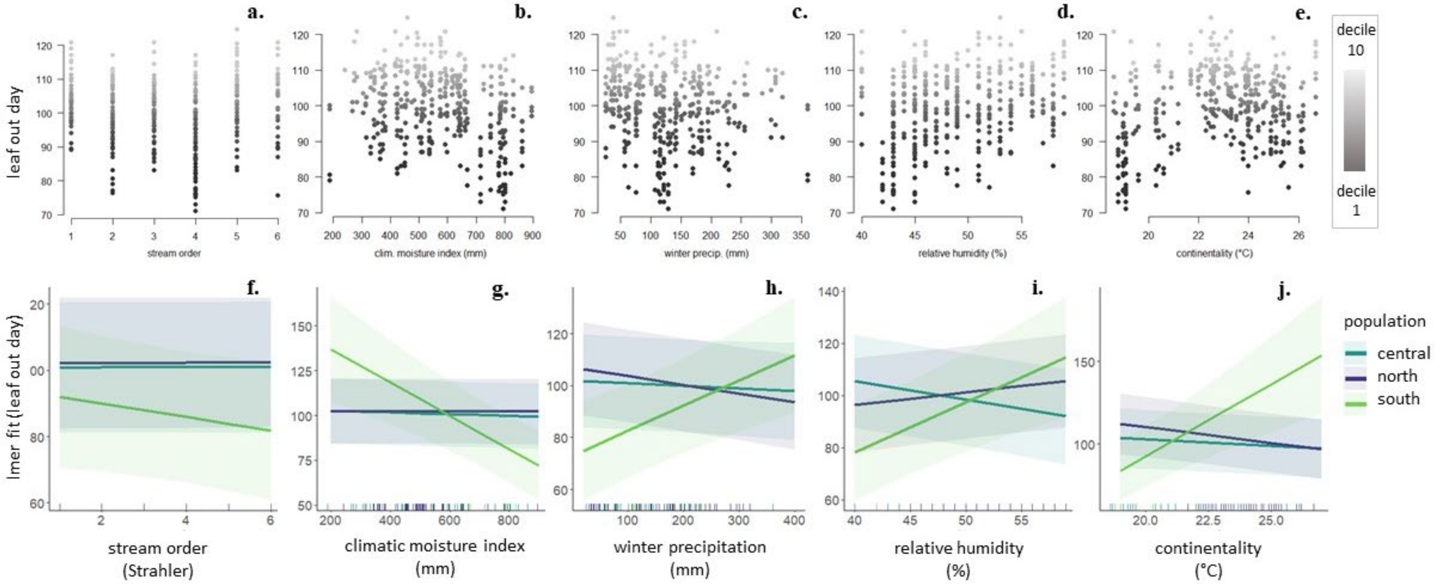

Supplementary Figure 3 (Fig. S3): Top row (panels a-e) represents the relationships between raw leaf out day and the environmental variables used in distribution models. **(a)** Strahler stream order, **(b)** Hargreave's climatic moisture index in mm, **(c)** winter precipitation in mm, **(d)** percent relative humidity, **(e)** continentality, measured as the difference between mean temperature of the coldest and warmest months in degrees Celsius. The greyscale represents the distribution of leaf out day values sorted into ten deciles. **Bottom row (panels f-j)** represents linear mixed effects model fits for the same variables in **(a-e)**, with population in the model as a random effect, and ribbons around each line represent upper and lower confidence intervals (0.95). Colors represent population.

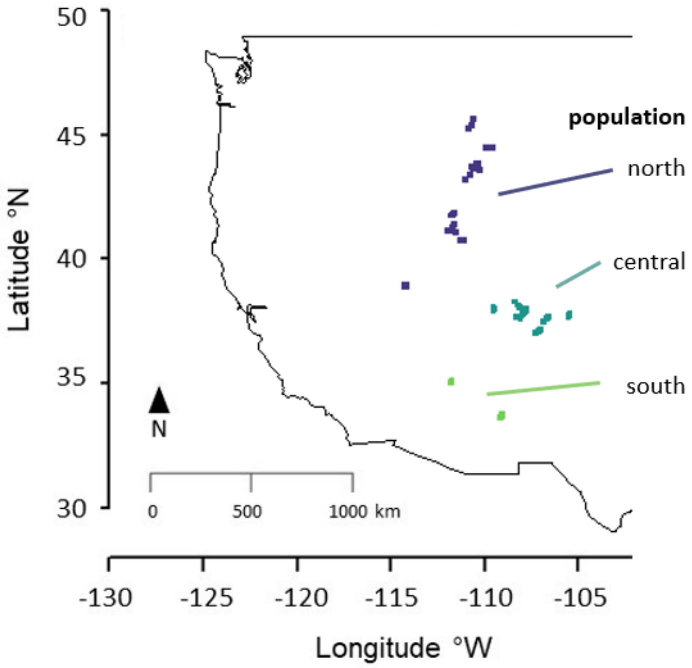

Supplementary Figure 4 (Fig. S4): Map of occurrence records (sampling locations) across the three genetic populations. Map is projected in WGS84 (World Geodetic System 1984) or EPSG 4326.

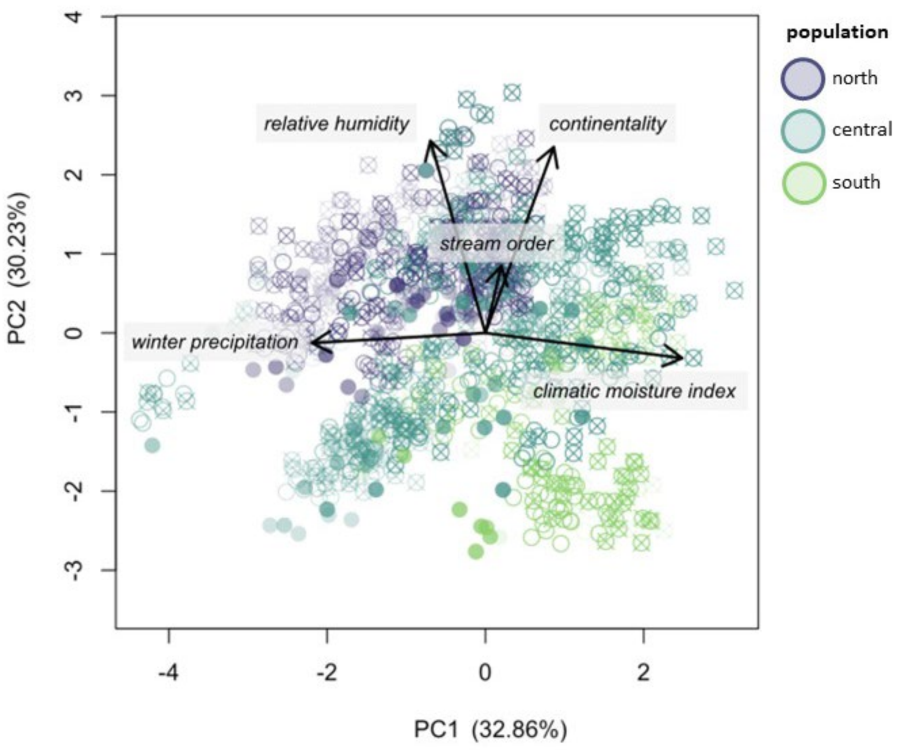

Supplementary Figure 5 (Fig. S5): Principal Coordinate Axes 1 and 2 of the five environmental variables used in distribution models. Different symbols represent different climatic scenarios (time, rcp, gcm). Color represents genetic provenance (purple= northern; teal= central; green= southern).
