# Supplementary material for: Stacked distribution models predict climate-driven loss of variation in leaf phenology at continental scales
Source: Commun Biol. 2022 Nov 10;5:1213. doi: 10.1038/s42003-022-04131-z (PMC9649771; doi:10.1038/s42003-022-04131-z)
Supplement: Supplementary file 2 — Description of additional supplementary files [file 42003_2022_4131_MOESM2_ESM.docx]

**Supplementary Data for:** Stacked distribution models predict climate-driven loss of variation in leaf phenology at continental scales

**Authors:** Shannon L.J. Bayliss*^1,2^, Liam O. Mueller^3^, Ian M. Ware^4^, Jennifer A. Schweitzer^1^, Joseph K. Bailey^1^

*Corresponding author; shannonljbay@gmail.com

Includes:

Descriptions of Supplementary Data 1-6

**File Name:** Supplementary Data 1

**Description:** Net predicted richness loss (Fig. 1) – map pixels (area) with negative (loss), zero, or positive (gain) change in richness across year, rcp, gcm, and genetic group.

**File Name:** Supplementary Data 2

**Description:** Precise predicted richness loss (Fig. 2) – map pixels (area) listed by predicted change in richness (ranges from -10 to 10) across year, rcp, gcm, genetic group.

**File Name:** Supplementary Data 3

**Description:** Average richness and similarity (Fig. 3) by year, rcp, gcm, genetic group.

**File Name**: Supplementary Data 4

**Description:** Average (of the 3 gcms) percent of landscape predicted suitable for the first and tenth trait deciles (Fig. 4) across time, rcp, and genetic group.

**File Name**: Supplementary Data 5

**Description:** Includes Maxent results (Fig. S2) of the percent variable contributions to decile models and training and testing AUC +/- standard deviation.

**File Name**: Supplementary Data 6

**Description:** Phenology data, latitude and longitudes of field occurrences, climate data used to build SDMs extracted at each tree’s field location at each year, rcp, and gcm (Fig. S3, S4, S5).
