# Supplementary material for: Stacked distribution models predict climate-driven loss of variation in leaf phenology at continental scales
Source: Commun Biol. 2022 Nov 10;5:1213. doi: 10.1038/s42003-022-04131-z (PMC9649771; doi:10.1038/s42003-022-04131-z)
Supplement: Supplementary file 9 — nr-reporting-summary [file 42003_2022_4131_MOESM9_ESM.pdf]

nature portfolio

Corresponding author(s): Shannon L. J. Bayliss

Last updated by author(s): 10/17/2022

Reporting Summary

Nature Portfolio wishes to improve the reproducibility of the work that we publish. This form provides structure for consistency and transparency in reporting. For further information on Nature Portfolio policies, see our Editorial Policies and the Editorial Policy Checklist.

Statistics

For all statistical analyses, confirm that the following items are present in the figure legend, table legend, main text, or Methods section.

n/a	Confirmed	
☐	☒	The exact sample size (*n*) for each experimental group/condition, given as a discrete number and unit of measurement
☐	☒	A statement on whether measurements were taken from distinct samples or whether the same sample was measured repeatedly
☐	☒	The statistical test(s) used AND whether they are one- or two-sided<br>*Only common tests should be described solely by name; describe more complex techniques in the Methods section.*
☐	☒	A description of all covariates tested
☐	☒	A description of any assumptions or corrections, such as tests of normality and adjustment for multiple comparisons
☐	☒	A full description of the statistical parameters including central tendency (e.g. means) or other basic estimates (e.g. regression coefficient) AND variation (e.g. standard deviation) or associated estimates of uncertainty (e.g. confidence intervals)
☐	☒	For null hypothesis testing, the test statistic (e.g. *F*, *t*, *r*) with confidence intervals, effect sizes, degrees of freedom and *P* value noted<br>*Give P values as exact values whenever suitable.*
☐	☒	For Bayesian analysis, information on the choice of priors and Markov chain Monte Carlo settings
☐	☒	For hierarchical and complex designs, identification of the appropriate level for tests and full reporting of outcomes
☐	☒	Estimates of effect sizes (e.g. Cohen's *d*, Pearson's *r*), indicating how they were calculated

Our web collection on statistics for biologists contains articles on many of the points above.

Software and code

Policy information about availability of computer code

Data collection	NHDPlus Version 2 (Reference 46)
Data analysis	ESRI ArcMap (2018) (Reference 47)<br>Maxent (Reference 51)<br>RStudio (R Core Team, 2022) (Reference 59)

For manuscripts utilizing custom algorithms or software that are central to the research but not yet described in published literature, software must be made available to editors and reviewers. We strongly encourage code deposition in a community repository (e.g. GitHub). See the Nature Portfolio guidelines for submitting code & software for further information.

Data

Policy information about availability of data

All manuscripts must include a data availability statement. This statement should provide the following information, where applicable:

- Accession codes, unique identifiers, or web links for publicly available datasets
- A description of any restrictions on data availability
- For clinical datasets or third party data, please ensure that the statement adheres to our policy

If not found referenced within the main manuscript or supplemental files, the datasets generated during and/or analysed during the current study are available

from the corresponding author on reasonable request.

Human research participants

Policy information about studies involving human research participants and Sex and Gender in Research.

Reporting on sex and gender	NA
Population characteristics	NA
Recruitment	NA
Ethics oversight	NA

Note that full information on the approval of the study protocol must also be provided in the manuscript.

Field-specific reporting

Please select the one below that is the best fit for your research. If you are not sure, read the appropriate sections before making your selection.

☐ Life sciences ☐ Behavioural & social sciences ☒ Ecological, evolutionary & environmental sciences

For a reference copy of the document with all sections, see nature.com/documents/nr-reporting-summary-flat.pdf

Ecological, evolutionary & environmental sciences study design

All studies must disclose on these points even when the disclosure is negative.

Study description	Species Distribution Modeling
Research sample	Populus angustifolia (narrowleaf cottonwood) sampled in 2012 in Western United States. Initial collection of 725 genotypes, 400 used in this study.
Sampling strategy	Sampling was designed to cover a wide range of environments across the species range and to collect multiple individuals across 3-5 sites along elevation gradients of >17 rivers (P. angustifolia is a riparian species). Sample size used in the study was the maximum number of individuals who survived in a greenhouse to have the relevant data collected.
Data collection	Data was collected in the field as latitude-longitude GPS data in WGS84 decimal values in 2012 and replicate cuttings were brought to a greenhouse and grown in common conditions. Greenhouse data used in the study was recorded in 2016, ~4 years after trees were collected from the field.
Timing and spatial scale	Described in "Data collection"
Data exclusions	No data was excluded.
Reproducibility	Methods section is written so that anyone can access the publicly available climate data used to build SDMs. Data to reproduce plots are included in the supplementary information. We have included a data availability statement and a code availability statement.
Randomization	Samples were split into three groups based on previous genetic analyses, referenced in the manuscript. Trees in the greenhouse were randomly spaced (i.e., not grouped by population or genotype) and were rotated periodically.
Blinding	Blinding was not relevant to this modeling study.

Did the study involve field work? ☒ Yes ☐ No

Field work, collection and transport

Field conditions	Field data used in this study were just latitude and longitude values, which are not dependent on climatic conditions at the time of collection.
Location	Sampling locations are represented in the manuscript figures. The western United States including the states of Wyoming, Utah, Colorado, Arizona, and New Mexico.
Access & import/export	No permits were needed for our collection sites.

Disturbance	No disturbances were caused by this study.

Reporting for specific materials, systems and methods

We require information from authors about some types of materials, experimental systems and methods used in many studies. Here, indicate whether each material, system or method listed is relevant to your study. If you are not sure if a list item applies to your research, read the appropriate section before selecting a response.

Materials & experimental systems

n/a	Involved in the study
☒	☐ Antibodies
☒	☐ Eukaryotic cell lines
☒	☐ Palaeontology and archaeology
☒	☐ Animals and other organisms
☒	☐ Clinical data
☒	☐ Dual use research of concern

Methods

n/a	Involved in the study
☒	☐ ChIP-seq
☒	☐ Flow cytometry
☒	☐ MRI-based neuroimaging
